# Has Tropical Babesiosis Always Been Endemic But Misidentified?

**DOI:** 10.4269/ajtmh.22-0178

**Published:** 2022-04-11

**Authors:** Sam R. Telford

**Affiliations:** Department of Infectious Disease and Global Health, Cummings School of Veterinary Medicine, Tufts University, North Grafton, Massachusetts

In this issue of the Journal, Gaur et al.^1^ report a case of human babesiosis from India. The asplenic patient sustained a 70% parasitemia, demonstrated clear signs of a hemolytic disease, became hypotensive after admission, and suffered a terminal cardiac arrest. *Babesia* sp. was identified from blood smears and by 18S ribosomal DNA (rDNA) sequencing. This is the second well-documented case of babesiosis from India; Marathe et al.^2^ previously provided a detailed case report, with photomicrographs of blood smears that established the diagnosis. That patient was a normosplenic 51-year-old man who presented with fever, palpable spleen, scleral icterus, and hemoglobinuria. Malaria was suspected, but “antimalarial drugs” had no effect. Once babesiosis was suspected, a standard course of clindamycin and quinine was followed by a rapid recovery.

The piroplasms (Hematozoa: Piroplasmida), comprising the genera *Babesia*, *Theileria* (including *Cytauxzoon*), *Anthemosoma*, and *Echinozoon*, are typically maintained by ticks and vertebrate hosts. It is likely that there are more than 100 valid *Babesia* spp. and perhaps a third as many *Theileria* spp. The piroplasms were initially thought to be very host specific, to the point that finding one in a different kind of animal prompted the description of a new species. Asa Chandler’s widely used introductory parasitology textbook^3^ had the pithy statement “they are found in all kinds of mammals *except* man.” The concept of host specificity of the *Babesia* was eroded when unequivocal evidence of human infection was first reported in 1957. Since then, two major epidemiologic patterns have been recognized: stable endemic risk due to *Babesia microti* in the northeastern and upper midwestern United States (incidence in highly endemic communities about 100 per 100,000 people/year), and sporadic global cases due to diverse *Babesia* spp., mainly those of ruminants. The diversity of piroplasms causing human babesiosis is increasingly recognized. Recently, an HIV-infected Zimbabwe resident suffering fever, anemia, and weight loss was found to be infected by *Anthemosoma garnhami*, a piroplasm of rodents^4^; despite the fact that the name of the causative agent is *Anthemosoma*, there is no need to apply a new name to the disease, which was typical of human babesiosis. To date, there has been no report of human infection by *Theileria* spp. or *Echinozoon*. Garnham and Bray^5^ failed to infect splenectomized chimpanzees by feeding *T. parva*-infected ticks on them, suggesting that *Theileria* spp. are unlikely to be found to infect humans.

Within the last two decades, *Babesia* spp. have been increasingly identified and reported from tropical, low- to middle-income countries, with classical morphologic and modern molecular confirmations. The question that remains to be answered is whether *Babesia* has been infecting people in tropical sites all along. Routine use of polymerase chain reaction (PCR), amplicon sequencing, and phylogenetic analysis has greatly promoted identifying etiologic agents, and our awareness of babesiosis as a potential rule out diagnosis has likely increased.

**Figure 1.**
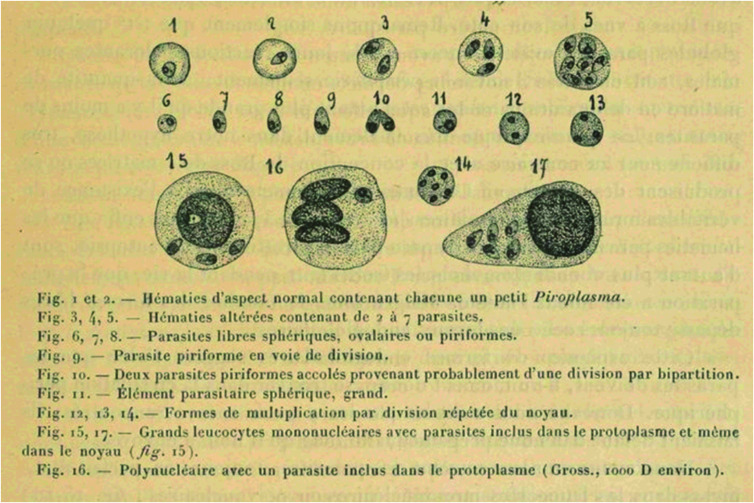
Laveran and Mesnil’s drawings from Donovan’s impression smears, in their description of “*Piroplasma donovani.*” Although their Figures 3, 9, and 10 are very suspicious, most of the other parasites shown appear to demonstrate a kinetoplast, which was surprisingly ignored by them. (A scan of the paper was provided via gallica.bnf.fr/BnF.)

It is not that tropical medicine workers have historically ignored the possibility of human babesiosis when searching for the etiology of fevers. Laveran and Mesnil^6^ named the agent of dum-dum fever *Piroplasma donovani*, after studying spleen impression smears from a cachectic Calcutta soldier sent to them by Donovan. They found piriform bodies within red blood cells, and indeed they looked tantalizingly like piroplasms (Figure 1). They wrote: “Les Piroplasmes occupaient deja une place importante en pathologie veterinaire. C’est las premiere fois qu’on signale une maladie humaine produite par un Piroplasme bien caracterise.” Ronald Ross,^7^ studying the same series of slides from Donovan as well as some sent to him by Leishman from a Madras case, opined that the parasites in such smears were artifactually superimposed on red blood cells, and noted the almost universal presence of a small rod of nuclear material perpendicular to a larger typical nucleus. He thus rejected the presence of a piroplasm, suggested the genus *Leishmania*, and reclassified the parasite as *Leishmania donovani.* An outbreak of “pyroplasmosis” in 1,800 of 2,500 residents of a village in Uttar Pradesh in India, notable for remittent fever that was not responsive to quinine, was reported in 1902.^8^ However, the illustrations of the causative parasites are more suggestive of *Plasmodium vivax*, and thus these cases do not comprise human babesiosis. Hayashi in 1906^9^ reported finding ring-shaped bodies in red blood cells at the eschars and within the viscera of tsutsugamushi disease patients and named “Theileria tsutsugamushi” as the cause. The rickettsial etiology of scrub typhus was suspected by 1918, and the agent named in the early 1930s. Similarities with redwater (bovine babesiosis) led Wright^10^ to report the involvement of a piroplasm in the development of blackwater fever, but the infecting organisms were later identified as plasmodia. It may be that many examples of erroneous incrimination of a piroplasm as an etiologic agent for a human disease inhibited future such considerations, leading to statements such as Chandler’s.

Although “piroplasm” implies pear-shaped, early babesial trophozoites generally assume a ring form. Given the morphologic similarity of piroplasm ring forms with those of *Plasmodium* spp., the prevailing hypothesis is that tropical babesiosis has been occurring all along, but with diagnoses missed, and the disease ascribed to malaria parasites. Distinguishing between *Babesia* spp. and *Plasmodium* spp. by microscopy can be difficult.

The eminent malariologist P.C.C. Garnham wrote in his authoritative monograph on the malaria parasites^11^ that seven validly described *Plasmodium* spp. (from dogs, otters, ruminants, porcupines, and even a snake) were in fact piroplasms, and that, when he had been asked to review slides of purported new plasmodia from animals, they usually turned out to be piroplasms. Indeed, the band-like and rosette forms shown by Gaur et al.^1^ are very suggestive of *P. malariae*, although the white vacuoles of many of the parasites are more consistent with *Babesia* spp. Garnham^12^ stated that the presence of these vacuoles were as useful as the absence of hemozoin in differentiating piroplasms from plasmodia. Dying babesia have prominent vacuoles (“crisis forms”^13^) and seem to be commonly represented in blood smears from animals or patients with heavy infections. The typical “Maltese cross” form (division into four merozoites) is seen only with piroplasms and establishes the diagnosis, but may not always be present in a blood smear.

The tropical population at risk is far greater than when babesiosis was first recognized as a zoonosis, and the global cohort of susceptible people has increased. There are 38 million people living with HIV/AIDS, and HIV is a known risk factor for *B. microti* babesiosis.^14^ Cancer patients are living longer; 60% of chronic myeloid leukemia patients in India are taking Glivec or imatinib.^15^ Rituximab, a known risk factor for treatment-resistant *B. microti* babesiosis,^16^ was placed on the WHO Model List of Essential Medicines in 2015. With increased life expectancy, there are more older individuals in the tropics. Babesiosis was first described by Smith and Kilborne in their classic investigations of Texas cattle fever as having age-related pathology, and that fact remains today.^17^ More susceptible people should imply more cases, even if transmission (ecologic) conditions have remained constant.

Garnham, who had reviewed slides and gave his opinion on the identity of the infecting parasites for four of the first eight known cases of human babesiosis,^12^ provides a quote that is still relevant today: “Man, particularly agricultural workers and people who spend much time in rural areas, undoubtedly comes into contact with Babesia-infected ticks quite frequently. Three species of piroplasms have been the cause of the three cases reported from human beings. Few places in the world are free of piroplasms; their presence presents a hazard to numerous people who are splenectomized and an unknown number whose splenic function is deficient.”^18^ Although ticks are said to be obligately required for transmission, *B. microti* is readily transmitted by blood and is the most common protozoal transmission risk associated with blood products in the United States.^19^ Humans exposed to animal blood may be at risk of acquiring infection without tick exposure. Nonetheless, it is unlikely that human babesiosis will be reported from sites where ticks are not common.

The vast majority of sporadic human babesiosis cases were initially detected by routine blood smear, with malaria the presumptive diagnosis. Modern molecular methods powerfully complement classical microscopy, additional analyses being stimulated by inconsistencies in clinical details and increased awareness of healthcare providers. Case reports such as that of Gaur et al.^1^ serve to educate clinicians and laboratorians in sites where human babesiosis has historically not been included in a differential diagnosis.
